# Allopathic Medicine Practitioners’ perspectives on facilitating disclosure of traditional medicine use in Gauteng, South Africa: a qualitative study

**DOI:** 10.1186/s12906-023-04270-8

**Published:** 2023-12-12

**Authors:** Lindiwe Gumede, Pauline B. Nkosi, Maureen N. Sibiya

**Affiliations:** 1 Department of Medical Imaging and Radiation Sciences, Faculty of Health Sciences, University of Johannesburg, Johannesburg, South Africa; 2 Department of Radiography, Faculty of Health Sciences, Durban University of Technology, Durban, South Africa; 3 DVC of Research, Innovation and Engagement, Mangosuthu University of Technology, Durban, South Africa

**Keywords:** Traditional medicine disclosure, Practitioner-patient approach, Treatment choice, Qualitative study

## Abstract

**Background:**

Traditional medicine (TM) plays a key role in maintaining health in many societies. Given the requirement for TM disclosure, Allopathic Medicine Practitioners (AMPs) must encourage open communication with patients to persuade those who use TM to disclose. Addressing patient non-disclosure of TM requires this dialogue to be facilitated. We sought to understand and describe how South African AMPs facilitate disclosure of TM use during a consultation with patients who use both TM and allopathic medicine (AM) and how it influences the patients’ willingness to disclose TM use.

**Methods:**

This qualitative exploratory descriptive study on AMPs at Gauteng district public hospitals in South Africa was conducted between 2021 and 2022. Non-probability purposive sampling was employed to select a sample of 14 AMPs. Individual participants were encouraged to share their unique experiences and interpretations of the phenomenon concerning TM use disclosure. The raw transcribed textual data were processed using ATLAS.ti, and inductive content analysis was undertaken following the coding of the content to identify categories.

**Results:**

The data revealed four major categories: ‘providing a suitable atmosphere for disclosure,’ ‘encouraging patients to disclose TM usage to AMPs,’ ‘patient autonomy,’ and ‘AMP training’. During a consultation with patients who use both TM and AM, participants expressed their experiences and perceptions of TM nondisclosure. They also discussed several methods for encouraging patients to disclose their TM usage, particularly when TM is used concurrently with AM.

**Conclusion:**

This study expands on previously reported findings by describing how South African AMPs facilitate the disclosure of TM use during consultation. Many AMPs struggle to initiate TM conversations with their patients which results in non-disclosure. This study revealed that integrating TM into AM training programmes, promoting cross-practice, and creating a safe environment is necessary for the development and application of the most appropriate approaches that would assist in facilitating disclosure.

**Supplementary Information:**

The online version contains supplementary material available at 10.1186/s12906-023-04270-8.

## Background

The history of the development of traditional medicine use dates to the Stone Age [1, 2] and is backed by archives of scientific observations that support its use [3]. The World Health Organisation’s (WHO) global report of 2019 confirms an increase in the number of member states that have a national TM policy and TM regulations in place from 1999 -2018 [4]. In addition, in three WHO global surveys between 2005 and 2018, 99 of 113 (88%) responding member states said their biggest challenge in TM was the need for more technical guidance on research and evaluation of the safety, efficacy, and quality of these treatments [4]. However, this is attainable if countries’ human resource capability for traditional medicine development is strengthened. As a result, educational systems should think about exposing health science students and professionals to the function of TM in health systems [5].

According to WHO (2013), 80% of the world’s population uses TM, and a majority of WHO member states, including South Africa, have reported using TM and requested help in creating a body of reliable research databases and data on TM practises and products. Sub-Saharan African nations generally acknowledge the use of TM, but most of the research is visible in nations like Nigeria, South Africa, Ghana, and Uganda [2]. In some communities, TM plays a crucial role in maintaining health [6]. In general, and because of the nature of their training, AMPs tend to view the usage of TM as a form of patient behaviour that is based on beliefs, which does not yield significant healthcare benefits, even when the practice continues despite a lack of access to healthcare services [7]. Since AMPs’ sceptical views on TM seem to be aligned with the Western paradigm of healthcare science and practices, decolonising the mindset regarding the nature and efficacy of TM, and the associated belief systems, may be necessary to change their views [8], as these views and attitudes may ultimately impact patient healthcare, especially concerning the disclosure of TM use [9]. There has been a significant global trend over the past 25 years to encourage the integration of TM use and personal care methods into recognised AM practices [10]. Because, if the TM used by patients who use both TM and AM is not disclosed, patients receive fragmented care which leads to subpar patient management [11].

Over the last decade, there has been minimal progress in TM disclosure rates since the nature of patient-provider communication has remained the primary influence [12]. A 2015 study [11] on TM use, treatment preferences, and AM substitution discovered that poor communication between AMPs and patients who use TM without disclosing it to AMPs is the primary cause of fragmented patient care. AMPs should encourage patients to disclose TM use, to lower interaction hazards between TM and AM while simultaneously building trust and encouraging greater disclosure throughout the consultation [13]. They also require a deeper understanding of TM to assist patients in disclosing their usage and to establish rapport to encourage honest responses [14].

Furthermore, they should create a conducive environment, AMPs should respect their patients’ beliefs about TM use and be aware of the implications of not disclosing its use during consultations [15]. Previous studies found that AMPs can facilitate this dialogue by asking direct questions which can elicit numerous reasons for non-disclosure [12, 16]. Undoubtedly, discussing their TM usage with the AMPs is crucial for the overall well-being of these patients. In addition, to improve the standard of healthcare for all patients, it is crucial to develop strategies for training AMPs to operate in ways that complement TM and respect various forms of knowledge [6, 17].

Organisations such as the World Health Organisation (WHO) are important in the development of strategies to improve healthcare worldwide. This organisation implemented a strategy (WHO TM Strategy 2014-2023) to help member states develop programmes that enhance the use of TM in healthcare, for those who want to implement them [2]. In addition, policymakers must make it easier for Traditional Health Practitioners (THPs) and AMPs, regulatory agencies, community organisations, and the public to communicate and work together [18]. This holds importance as there are now policies in place that incentivise THPs to officially register their practices [19]. However, there is a considerable amount of research indicating that patients’ reluctance to reveal information to their healthcare providers is heavily influenced by their perceptions of their AMPs [14, 20]. Therefore, patients should be involved in the various issues that concern their healthcare and receive clear information about recommended treatments as well as alternative treatment options [21].

In South Africa, Allopathic Medicine Practitioners are required to perform six essential functions [22]. These functions comprise providing care, serving as a consultant, building capacity, training in clinical settings, leading clinical governance, and advocating for community-oriented primary care. AMPs are aware of the possibility that their patients are using TM when performing these essential roles; as a result, disclosure of TM use during a consultation must be encouraged [23, 24], but AMPs detach themselves from this issue when performing their roles [25]. Consequently, patients conclude that disclosure of TM use is unimportant to AMPs when AMPs do not enquire about TM use [25].

Allopathic Medicine Practitioners must also demonstrate a significant commitment to learning about the use of TM and this can be achieved by, among other things, asking pertinent questions about its use whenever they consult with patients who use TM [26]. Fischer and Ereaut [27] presented an idea for creating a consultation model, which they liken to a situation where the AMP and the patient are dancing together but only the AMP is aware of the music and the steps. However, this consultation model does not provide how patients can disclose during consultation. To close this gap, the current study may shed light on how AMPs could facilitate disclosure of TM use during a consultation with patients who use both TM and AM.

## Methods

### Study design

The study used a qualitative exploratory, descriptive study design to explore facilitating disclosure of TM use during a consultation with patients who use both TM and AM. It seeks to comprehend a phenomenon in its natural setting by studying an individual’s perspectives and experiences [28]. Exploratory research creates new presentation strategies and fundamental approaches whereas descriptive research documents a phenomenon and develops effective interventions [29]. One-on-one, semi-structured interviews were used to explore how AMPs facilitate disclosure of TM use and motivated the AMPs to detail their experiences in facilitating disclosure of TM use. An interview guide (Supplementary file 1) containing open-ended questions was used.

### Sampling procedure and setting

The population of interest included qualified AMPs also known as Physicians working at selected district public hospitals in Gauteng province. Allopathic Medicine Practitioners oversee and manage primary care health services at district hospitals, community health centres (CHCs), and PHC clinics [30]. After obtaining permission from the selected institutions, the primary researcher initiated the recruitment process. To ensure the exploration of the facilitation of TM use disclosure, the researchers recruited samples from multiple sites [29]. Fourteen AMPs (*n*=14) sampled through non-probability purposive sampling participated in the study. The authors (L.G, P.B.N. and M.N.S.) assume that most AMPs need to facilitate disclosure during a consultation with patients who use both TM and AM. The AMPs had various demographics. Therefore, the sample population was restricted to AMPs who are presently in practice and who possess particular characteristics. The inclusion criteria were considered if the participants were registered with the Health Professionals Council of South Africa (HPCSA), and permanently employed at the hospital with a minimum of 1 year of experience in general patient management. Therefore, all medical interns, registrars, AMPs on community service and retired AMPs were excluded from the study because they are not always accessible and accountable for ensuring progress in improving quality healthcare. In South Africa, AMP training includes interns completing two years of training at an accredited institution under close supervision and rotation through various medical specialities and departments, followed by a year of community service during which they start working on their own [31] on a contract basis. While registrars have the experience required for inclusion in the study, their training consists primarily of rotations between Public Health Clinics and public hospitals and therefore, they are not allocated to a single institution during training [32] and retired AMPs are not usually hired regularly in these hospitals as they are usually hired on a contract basis.

The study was conducted in the outpatient departments at the selected public hospitals in Gauteng Province. Gauteng contains three metropolitan municipalities (the City of Johannesburg, Ekurhuleni, and Tshwane) and two district municipalities, which are further subdivided into six local municipalities [33]. This study took into account the hospitals’ diverse surroundings. District hospitals are level 1 hospitals in Gauteng, and selected AMPs (physicians) practice there. This study selected district hospitals because they promote primary care and provide access to more specialised treatment. Besides, these Gauteng hospitals were selected because they are in the most populous districts. This ensured population representation in the study area.. These hospitals treat most chronic patients who use TM and AM. Only three Gauteng districts, namely the City of Johannesburg Metropolitan District, Ekurhuleni Metropolitan Municipality, and Tshwane Metropolitan Municipality, were studied. Then, 4 district hospitals from the three areas were sampled. Public health clinics refer patients to district hospitals for outpatient services.

### Data collection

From November 2021 to July 2022, qualitative information was gathered via in-depth individual interviews. All eligible participants were approached face-to-face and given an information letter and consent form for consideration. Appointments were set up with the primary researcher for those who were interested. The primary researcher (L.G) conducted one-on-one interviews with all participants who voluntarily consented to participate in the study, adhering to the necessary COVID-19 restrictions. LG is a South African woman with a background in diagnostic radiography. She was a PhD candidate at the time of this study. She currently holds a PhD in Health Sciences and has experience conducting semi-structured interviews with South African health professionals. All authors are employed at various higher education institutions in South Africa. L. G is a lecturer at the University of Johannesburg in Gauteng province, P.B.N. is a Senior lecturer at the Durban University of Technology in Kwazulu Natal province and M.N.S. is the Deputy Vice-Chancellor for research, innovation, and engagement at the Mangosuthu University of Technology in Kwazulu Natal province.

The study utilised a pre-tested semi-structured interview guide for gathering information on AMPs’ perceptions of facilitating disclosure of TM use. An interview refinement protocol (IPR) framework was used [34], and the guide was approved by the second (P.B.N) and third (M.N.S.) authors who both have PhD in Health Sciences and D. Tech in Nursing, respectively and the institutional ethics committee. Pilot interviews were conducted [35] with two individuals to familiarise the primary researcher with the procedure. All the interviews were audio-recorded with participants’ permission. Prompts were used to confirm and clarify opinions [36], allowing the primary researcher to identify patterns, emergent categories, and sub-categories. Each interview lasted for approximately 30 – 45 minutes. Field notes were made during each interview and included in the final data analysis to track observations. Data saturation was observed when interviews did not yield new information.

### Ethical considerations

Ethical approval was obtained from the Durban University of Technology Institutional Research Ethics Committee (IREC 016/21). Before the commencement of the interviews, the research objectives were communicated and explained to the participants through the information letter delivered by LG. Informed consent was obtained before data collection. All information about the research was kept confidential and private in a password-protected computer. In compliance with Act 4 of 2013 on the Protection of Personal Information (POPI), only the researchers had access to the information [37]

### Data analysis

**Fig. 1:**
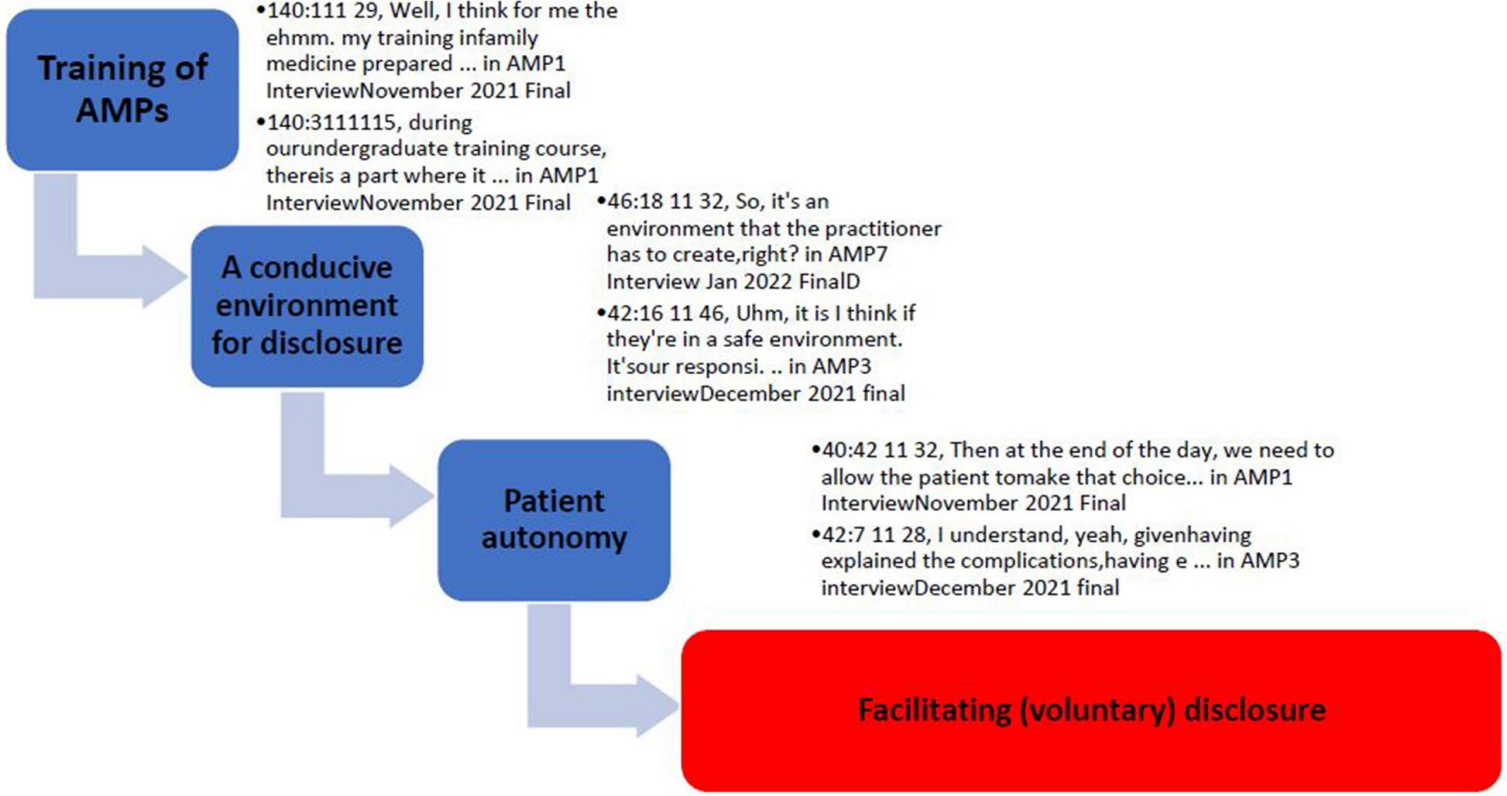
The coding tree diagram depicts quotes and codes that form the categories

Data analysis was initiated by the primary researcher transcribing three transcripts and sending them to P.B.N and M.N.S. for observation to ensure high-quality data retrieval and to mitigate bias [38]. The primary researcher transcribed and validated the subsequent transcripts, which were then uploaded to ATLAS.ti-9 for data coding. To enable the authors to develop a conceptual understanding through interpretation and explanation, data were further subjected to inductive content analysis [39]. Inductive content analysis was conducted to develop a conceptual understanding through interpretation and explanation. All transcripts were reviewed and reread, and thoughts were categorised based on the dataset’s content. The connected categories are illustrated in Fig. 1 as a coding tree diagram.

The first categories were arranged based on how the words captured the meaning of the data. The analysis of the initial categories led to the formation of major categories and sub-categories. Adhering to the social constructivism methodological approach ensured accurate interpretations of the lived experiences of AMPs who consulted with patients using both TM and AM [40]. This provided a general picture of how non-disclosure may have affected consultation with patients using both TM and AM, highlighting similarities and differences between AMPs’ experiences.

To the ensure trustworthiness of the data analysis, four standard criteria: credibility, dependability, confirmability, and transferability were used [41]. To confirm that the processing, analysis, and interpretation of data accurately reflected the reality of occurrences of disclosure or non-disclosure of TM use by patients as experienced by the AMPs [40], credibility was verified through researcher triangulation [38]. Giving detailed specifics about the study’s methodology contributes to making the findings more understandable and reliable. To ensure completeness and veracity, the researcher documented all data collected during the interviews and verified whether the researcher’s interpretations and comprehension of the concepts identified through data analysis corresponded to the participants’ opinions and experiences during the participants’ debriefing. By interviewing AMPs with a range of experiences, the researchers were able to offer adequate background descriptions to answer the research question.

## Results

This study is a component of a wider investigation that aimed at developing the guidelines for disclosing TM usage to AMPs at hospitals in Gauteng, South Africa. During the study, 14 AMPs were interviewed. Table 1 displays the demographic and specified occupational information of the participants. This study adhered to the Consolidated Criteria for Reporting Qualitative Research (COREQ), details as illustrated in Supplementary file 2. To highlight the anonymised opinions of AMPs for privacy, we selected typical excerpts labelled with pseudonyms. Facilitating disclosure of traditional medicine use to AMPs was divided into 4 major categories and 7 sub-categories displayed in Table 2.

**Table 1:** Demographic characteristics of AMPs (*n*=14)

Characteristics	Variables	n (%)
*Sex*	Female	7 (50%)
	Male	7 (50%)
*Age group (Years)*	28 - 40	3 (21.4%)
	41 - 50	6 (42.8%)
	50+	5 (35.7)
*Race*	Black	13 (92.8%)
	Indian	1 (7.1%)
*Marital Status*	Single	4 (28.5%)
	Married	10 (71.4%)
*Qualification*	Undergraduate	13 (92.8%)
	Postgraduate	1 (7.1%)
*Work experience (years)*	3 – 5	2 (14.2 %)
	6 - 10	2 (14.2 %)
	11 – 15	4 (28.5%)
	16 – 20	3 (21.4%)
	21 - 30	1 (7.1%)
	30+	2 (14.2 %)

**Table 2:** Categories and sub-categories

Categories	Sub-categories
Creating a conducive environment for disclosure.	A safe environment to disclose
	Free and comfortable disclosure
Encouraging patients to disclose TM use to AMPs.	Asking non-prejudiced direct questions
	Provision of information to the patient
Patient autonomy.	Supporting patient’s choice
	Patients’ right to choose treatments of their choice
Training of AMPs.	Trained mainly in the use of AM

### Category 1: Creating a conducive environment for disclosure of TM use

#### A safe environment to disclose.

Participants in this study acknowledged that AMPs have to create a welcoming environment for all patients, and they demonstrated awareness of this obligation. Many showed a desire to make the patients feel welcome to encourage disclosure. They claimed that regardless of their position on TM usage, having a uniform mode of approach for all patients would help to ensure that patients who use TM would disclose.

“Uhm, I believe a patient discloses in a safe environment. It is our responsibility as healthcare providers to create a safe environment in which patients can disclose anything, even if it is not traditional medicine. However, whatever adjuvant treatment they are receiving, we must create a safe environment.” [AMP3]

“Patients, in my opinion, should be required to disclose. I believe that practitioners must keep an open mind and be able to create an environment in which the patient can speak. So, does the practitioner have to create an environment?... First, the environment must be clear and conducive, and trust must be established by you (the AMP). It is the practitioner’s responsibility to create that platform to clarify the implications of the patient’s illness and treatment. Practitioners must foster an environment in which patients are encouraged to disclose.”[AMP7]

#### Free and comfortable disclosure

To ensure effective communication between AMP patients and to foster a comfortable environment of trust, disclosure during a consultation with AMPs must not be restricted. According to the participants, patients would be unwilling to disclose anything beyond what they believed was necessary to receive assistance at the time if they did not feel accepted in the environment. Most participants indicated that a safe environment would instil within the patient some sense of trust towards the person they were interacting with.

“… Ok. First, when I consult with a patient, I want the patient to feel safe and confident; when the patient is with me, I want the patient to trust me because I’m here to help, not to harm; and once the patient trusts you (the AMP), I believe it will be easy for the patient to disclose.” [AMP4]

“Uh, we’d like patients to fully disclose because we don’t want them to be hiding anything from us. So, we’d like to provide an environment in which a patient feels very safe to disclose and is not judged by whatever treatment they’re using, but ultimately, we’d like patients to give us full disclosure about what they’re using because it affects how we manage them further.” [AMP13]

### Category 2: Encouraging patients to disclose TM use to AMPs

#### Asking non-prejudiced direct questions

Participants expressed that patients could be encouraged to reveal if they were properly communicated with and using the right approach. They claimed that patients would readily disclose their use of TM during a consultation if they could see that other patients were aware of it. They suggested that even if a patient disputed responsibility for their circumstances, they still needed to feel confident that they could contact the AMPs and trust that they would handle the disclosure effectively.

“… However, if you ask the patient directly, they will not disclose unless they believe you [the AMP] are receptive. If I’m curious, I’ll tell the patient that most of my patients take traditional medications. So, when they suspect that they are not the only ones using traditional medicine, they reveal it. But if I simply asked, they would not reveal.” [AMP1]

“We (the AMPs) should not judge patients for their own choices to allow the patient to disclose. We must recognise that patients have rights. We should also inform patients that their rights entail responsibilities. We [the AMPs] also have a responsibility to protect patients.” [AMP3]

“... In my opinion, I want the patient to disclose because I want to know everything about the patient when I’m treating him or her. As a result, if the patient discloses, we should always enquire as to how long this has been going on. Is there anything else wrong with them? And what other medications are they taking? You’ll discover that some medication could be the source of the patient’s symptoms?” [AMP4]

#### Provision of information to the patient

Participants believed that providing the patient with pertinent information could result in effective treatment. They believe that patients would get the intended benefit from the treatment and help them avoid adverse reactions. Some participants thought that these patients would be capable of making informed decisions regarding the use of TM if they had access to enough information.

“OK, so they would disclose, one, if they are aware of information that would benefit them. So, having explained the preceding point, they should be allowed to fully express themselves. They should be aware of the impact of their treatment plans on their condition, as well as the interaction of their treatment plans with other treatments that they may be using. As a result, if they are aware of such information, it will be easier for them to disclose.” [AMP13]

“For the patient to disclose, I usually ask the patient. I ask the patient to tell me the truth, and when I discover they are using traditional medicine, I don’t dismiss them; instead, I speak appropriately. I show them that traditional medicines in this condition are not to be used because they will all harm your kidneys.” [AMP9]

“So, when patients come to us, uh [AMPs], uh, I’d usually ask them that, but I’d also give them information about our treatment plan, and they’d decide which plan they wanted to go with on their own.” [AMP13]

### Category 3: Patient autonomy

#### Supporting Patients’ Choice

Participants agreed that patients who use TM and AM concurrently cannot be pressured just to use AM if they believe TM to be useful for them. They felt that it was necessary to support patients in choosing the treatments that best suited them.

“.... I don’t mind if there are no negative interactions between traditional medicines and our allopathic medicine.” [AMP2]

“For example, in traditional medicine, let’s say a patient comes in for an acute illness and they don’t necessarily have any chronic conditions, and I have no reason to suspect any other chronic illnesses. I don’t usually have a problem with that (referring to the use of TM).” [AMP5]

“Uhm…We [AMPs] have a responsibility to make sure that we are always constant, and the patient autonomy is respected.” [AMP3]

“I used to be autocratic before I went to get my speciality, but not anymore. I no longer use that method; instead, I simply allow the patient to make their own decision because, at the end of the day, it is the patient’s choice. I can’t make the patient do anything.” [AMP1]

“…The patient must consider the outcomes. However, it is ultimately the patient’s choice. I can’t force it.” [AMP4]

“I always keep an open mind when dealing with patients who have different perspectives on medical treatment. So, if a patient has a different point of view. I’m always willing to sit down with a patient and talk about it [the TM use].” [AMP7]

#### Patients’ right to choose treatments of their choice

Participants argue that patients should be softly persuaded while being shielded from any attempts at control. They recommended that patients be allowed to take responsibility for their behaviour because if they felt that they were being controlled or coerced, they might not feel responsible for the recommendation made and would not feel liable for any consequences. Participants thought that the patients should use their rights without fear of reprisal. Soft skills in communicating towards disclosure are as asset, thus:

“We should not pass judgment on patients based on their choices. We must recognise that patients have the right to choose their treatments. We should also remind patients that their rights come with responsibilities. On the other hand, we have a responsibility to protect the patients and maintain our commitment to patient autonomy.” [AMP3]

“Yeah, well, we should talk properly. They [the patients] are of legal drinking age. They have the right to make their decisions. They have the right to know as well. The more information we provide, the clearer their decisions will be.” [AMP9]

### Category 4: Training of allopathic medicine practitioners

#### Trained mainly on the use of allopathic medicine

Participants stated that AMPs are trained to consider other complementary methods of treating the patient. However, they also mentioned that TM was not considered an alternative to AM, except in instances where the individual AMP had previous exposure to TM use. They also mentioned that traditional health practitioners (THPs) could also benefit from health education as this could encourage their visibility as people who are involved in patient treatment.

“…. It is our training that will make us opposed to the use of traditional medicine.” [AMP2]

“...We [AMPs] were primarily trained in the use of a medical treatment" [AM]. So, our approach would be for the patient to use what we recommended. As a result, my opinion strongly supports what I know about the medical treatment aspect. As a result, we usually advise patients to use medical treatment rather than other treatment options about which we are unsure.*”* [AMP13]

“But our medical training does not prepare us for this type of scenario [referring to patients who use TM] because you are always told to do what is best for the patient, but this is not always the case... However, during their undergraduate training, doctors are exposed to technology that does not allow us to know what they [THPs] are doing; we simply assume they throw bones [a method of checking with the ancestors to determine what problems the patient may be experiencing] and then [showing a gesture of bare hands]. There is very little understanding of what occurs on the other side.” [AMP1]

“…. if I find out the patient is using traditional medicine, as a medical practitioner, I don’t have a clue about traditional medicine.” [AMP12]

## Discussion

The majority of research studying TM disclosure has highlighted the scarcity of literature on AMP perceptions of TM disclosure. These include previous studies from England [42] Taiwan [43], Norway [24], Australia [12] and Malaysia [15]. The majority of these studies highlighted concerns with AMP patient enquiries and the impact of non-disclosure on the administration of proper health care to TM patients. [12, 15, 24, 42, 43]. To provide patients with the healthcare system they deserve, a dialogue encouraging collaboration with THPs must be initiated [44]. Therefore, “decolonising” medicine may be necessary which involves “humanising” medicine so that counter-colonial stories that are genuine to patients that use TM can emerge and we can begin to understand health, illness, and healing [45].

Traditional medicine, provides patients with simple access to treatment, allowing them to collaborate in making their own health decisions and resulting in enhanced patient autonomy [46]. According to available evidence from this study, AMP acceptance of TM and allowing it to be freely addressed in a non-judgemental manner may develop trust and enable disclosure to promote safe and dependable treatments for patients [47, 48]. Other studies have found that patients who take both TM and AM should reveal their TM use to improve their overall treatment outcomes [49, 50]. AMPs, on the other hand, should embrace their patients’ decisions to use TM and endeavour to give them all of their available options because they, too, are people with diverse perspectives [51]. In light of this, patients should have access to information about the TM they use to exercise their choice, as this is crucial to ensure the quality of TM since this is an important issue globally [52].

However, as an initial step, TM should be integrated into the training programme of AMPs, there should be cross-practice between AMPs and THPs and recommendations for highly regarded TM in health facilities should be made [48, 53, 54]. Furthermore, AMPs should actively enquire about the use of TM, but this can only be achieved through the inclusion of TM education in the AM curricula to develop the AMPs’ ability to initiate the discussion of TM use during the consultation [55]. Therefore, the acquired knowledge of TM and the relationships between TM and AM among patients and AMPs would enable AMPs to be more open with their prescriptions and involve patients in their care [55].

Facilitating disclosure of TM use presents AMPs and patients who use TM with both unique challenges and significant opportunities. AMPs have a responsibility to ensure that all patient consultations take place in a conducive setting where the patient can divulge any additional information that might help with treatment planning. Creating a safe environment for the patient and demonstrating empathy can promote open communication and increase the likelihood of disclosure [14]. Because non-disclosure is so common, it is necessary to create an inclusive healthcare system supported by AMPs who can interact with different patient types [56]. Therefore, AMPs should be aware of the barriers to the disclosure of TM and endeavour to improve their interactions with these patients [57].

Nonetheless, this study identified that AMP training does not incorporate TM information as a key barrier to promoting patient disclosure of TM usage. This finding contradicts a recent study which includes South Africa as one of the countries in Sub-Saharan Africa that provide formal TM education [58]. Other research has reported AM students training at various universities in South Africa requesting training that includes teaching about the fundamentals of TM, how to empathise with patients who use TM, and how to approach such patients during a consultation [59]. Furthermore, it is acknowledged that the solution entails not only modifying curricula and student understanding but also changing faculty mindsets in this regard [59].

## Recommendations

It may not be possible for AMPs in their institutions to change some of the variables impacting the AMPs’ impressions of TM, such as the scepticism of THP training. However, TM should be incorporated into AMPs’ training programmes, there should be cross-practice between AMPs and THPs, and suggestions for highly valued TM in health institutions should be made to legitimise and justify TM in terms of practice and knowledge. The development of scaffolded TM and AM training gradually integrates AMPs into an understanding of TM with a focus on both increasing their knowledge and identifying primary causes of perceived TM effects so that AMPs can be informed about many issues regarding TM. AMPs could better achieve their treatment objectives by encouraging patients who use both TM and AM to disclose their use of TM.

It is probably critical to look for methods to make disclosure of TM use to AMPs easier. Future studies may investigate the relationship between AMP training and patients who use both TM and AM without disclosing their TM use. In addition, they may be studies on the perspectives of patients who use both AM and TM in the African setting. Furthermore, research on specific recommendations for how AMPs could improve the TM disclosure process is critical.

### Strengths and limitations of the study

Debatably, this study is the first comprehensive one on facilitating the disclosure of traditional medicine to AMPS in South Africa. The research focused on AMPs, who were informed about the non-disclosure of TM used by patients using both TM and AM. Participants shared their training expertise and experiences to improve disclosure. However, the study did not investigate how AMPs would protect patient confidentiality, which is crucial for healthcare practice. The sample was predominantly black AMPs, which might have constrained the study’s understanding of social attitudes towards TM use. The results were limited to the population under consideration thus and cannot be generalised to other provinces in South Africa.

## Conclusion

This study examined the opinions of AMPs on TM disclosure and its current shortcomings. It found that patients’ motivation to disclose TM use and decision-making process varies with AMPs. Factors contributing to these variations include establishing a transparent setting, encouraging patients to be forthcoming about their use of TM, and training AMPs for such encounters. The current AM practice lacks space for diverse perspectives, and the power hierarchy should be abolished. Safe spaces within AM training can facilitate productive discussions about TM integration in the healthcare system. AMPs may feel pressured to treat patients who use both TM and AM without confirmation of TM use. More guidance is needed on addressing factors influencing patients’ willingness to disclose during consultations and ensuring flexible TM disclosure processes. The study recommends incorporating TM training into all AMPs’ training curricula. In response to the study findings, guidelines will be developed to facilitate TM disclosure to AMPs, and standardised disclosure processes for all consultations will promote understanding and patient involvement.

### Supplementary Information

**Additional file 1.** Interview questions.

**Additional file 2.** Consolidated criteria for reporting qualitative studies (COREQ): 32-item checklist.

## Data Availability

The evidence that supports the findings provided in this study is available from the corresponding author as anonymised raw data upon good justification. Because of ethical issues about the protection of participant information, data are not publicly available. In addition, the Allopathic Medicine Practitioners who took part in the study did not consent to their data being released publicly. Furthermore, the Durban University of Technology Institutional Research Ethics Committee and the participating hospitals in Gauteng Province did not approve of the data being made public.
