# Supplementary material for: Allopathic Medicine Practitioners’ perspectives on facilitating disclosure of traditional medicine use in Gauteng, South Africa: a qualitative study
Source: BMC Complement Med Ther. 2023 Dec 12;23:451. doi: 10.1186/s12906-023-04270-8 (PMC10717688; doi:10.1186/s12906-023-04270-8)
Supplement: Supplementary file 1 — Additional file 1. Interview questions. [file 12906_2023_4270_MOESM1_ESM.docx]

**Authors:** Lindiwe Gumede^1^*

Pauline B. Nkosi^2^

Maureen N. Sibiya^3^

**Affiliation:** ^1^Department of Medical Imaging and Radiation Sciences, Medical Imaging and Radiation Sciences University of Johannesburg, South Africa.

^2^Department of Radiography, Faculty of Health Sciences, Department of Radiography Durban University of Technology, South Africa.

^3^DVC of Research, Innovation and Engagement, Mangosuthu University of Technology, South Africa.

**Interview Guide for AMPs**

**INTERVIEW QUESTIONS**

1. During consultation, how do you deal with patients that present with views that are different than yours regarding medical treatment?
2. In your opinion what will make a patient who use both TM and AM to disclose TM use?
3. What recommendations will you provide that will encourage the disclosure of TM use to AMPs by patients who use both TM and AM?

**Probing questions will be guided by participants’ responses during the interview session.**

**The following questions are intended to help the interviewer probe deeply about the issue at hand.**

- What does this remind you of?
- What would need to change in order for you to accomplish this?
