# Supplementary material for: Allopathic Medicine Practitioners’ perspectives on facilitating disclosure of traditional medicine use in Gauteng, South Africa: a qualitative study
Source: BMC Complement Med Ther. 2023 Dec 12;23:451. doi: 10.1186/s12906-023-04270-8 (PMC10717688; doi:10.1186/s12906-023-04270-8)
Supplement: Supplementary file 2 — Additional file 2. Consolidated criteria for reporting qualitative studies (COREQ): 32-item checklist. [file 12906_2023_4270_MOESM2_ESM.docx]

**Manuscript: Allopathic Medicine Practitioners’ perspectives on facilitating disclosure of traditional medicine use in Gauteng, South Africa: A qualitative study**

**Consolidated criteria for reporting qualitative studies (COREQ): 32-item checklist.**

Developed from:

Tong A, Sainsbury P, Craig J. Consolidated criteria for reporting qualitative research (COREQ): a 32-item checklist for interviews and focus groups. *Int J Qual Health Care*. 2007; 19(6): pp. 49 – 357. <http://intqhc.oxfordjournals.org/content/19/6/349.long>

| **No. Item** | **Guide questions/description** | **Reported on Page #** |
| --- | --- | --- |
| **Domain 1: Research team and reﬂexivity** |  |  |
| *Personal Characteristics* |  |  |
| 1. Interviewer/facilitator | Which author/s conducted the interviews? | Page 5 / paragraph 15 (Line 179) |
| 2. Credentials | What were the researcher’s credentials? e.g., PhD, D.tech | Page 5 / paragraph 15 (Line 182)  Page 5/ paragraph 15 (line 193) |
| 3. Occupation | What was their occupation at the time of the study? | Page 5 / paragraph 15 (Line 184-188) |
| 4. Gender | Was the researcher male or female? | Page 5 / paragraph 15 (Line 181) |
| 5. Experience and training | What experience or training did the researcher have? | Page 5 / paragraph 15 (Line 182) |
| *Relationship with participants* |  |  |
| 6. Relationship established | Was a relationship established prior to study commencement? | No, the researcher was not known to participants |
| 7. Participant knowledge of the interviewer | What did the participants know about the researcher? e.g., personal goals, reasons for doing the research | Page 5 / paragraph 17 (Line 207)  **All eligible participants were given an information letter and consent form consideration and those who were interested were arranged appointments with the primary researcher at a time convenient to them.** |
| 8. Interviewer characteristics | What characteristics were reported about the inter viewer/facilitator? e.g., Bias, assumptions, reasons, and interests in the research topic | Page 6 / paragraph 19 (Line 212-213) |

| **Domain 2: study design** |  |  |
| --- | --- | --- |
| *Theoretical framework* |  |  |
| 9. Methodological orientation and Theory | What methodological orientation was stated to underpin the study? e.g., grounded theory, discourse analysis, ethnography, phenomenology, content analysis | Page 4 /paragraph12 (Line 127) |
| *Participant selection* |  |  |
| 10. Sampling | How were participants selected? e.g., purposive, convenience, consecutive, snowball | Page 3 / paragraph 13 (Line 144) |
| 11. Method of approach | How were participants approached? e.g., face-to-face, telephone, mail, email | Page 5/ paragraph 17 (Line 198) |
| 12. Sample size | How many participants were in the study? | Page 4 / paragraph 13 (Line 177) |
| 13. non-participation | How many people refused to participate or dropped out? Reasons? | **None** |
| *Setting* |  |  |
| 14. Setting of data collection | Where was the data collected? e.g., home, clinic, workplace | **Hospitals (Research sites)** |
| 15. Presence of non-participants | Was anyone else present besides the participants and researchers? | **No** |
| 16. Description of sample | What are the important characteristics of the sample? e.g. demographic data, date | Page 7 / (Line 244 and 257) |
| *Data collection* |  |  |
| 17. Interview guide | Were questions, prompts, and guides provided by the authors? Was it pilot tested? | Page 3/ paragraph 16 (Line 190-191) |
| 18. Repeat interviews | Were repeat interviews carried out? If yes, how many? | **No** |
| 19. Audio/visual recording | Did the research use audio or visual recording to collect the data? | Page 5 / paragraph 15 (Line 195)  **Audio** |
| 20. Field notes | Were ﬁeld notes made during and/or after the interview or focus group? | Page 5 / paragraph 16 (Line 198)  **Field notes** |
| 21. Duration | What was the duration of the interviews or focus groups? | Page 5 / paragraph 16 (Line 198) |
| 22. Data saturation | Was data saturation discussed? | Page 5 / paragraph 16 (Line 199) |
| 23. Transcripts returned | Were transcripts returned to participants for comment and/or correction? | **No** |

| **Domain 3: analysis and ﬁndings** |  |  |
| --- | --- | --- |
| *Data analysis* |  |  |
| 24. Number of data coders | How many data coders coded the data? | **3 (Main researcher and 2 authors)** |
| 25. Description of the coding tree | Did authors provide a description of the coding tree? | Page 6 / paragraph 20 (Line 255)  Supplementary file 3 |
| 26. Derivation of themes | Were themes identiﬁed in advance or derived from the data? | Page 4 / paragraph 13 (Line 215-219)  **Derived from the data.** |
| 27. Software | What software, if applicable, was used to manage the data? | **ATLASti.9** |
| 28. Participant checking | Did participants provide feedback on the ﬁndings? | **No** |
| *Reporting* |  |  |
| 29. Quotations presented | Were participant quotations presented to illustrate the themes/ﬁndings? Was each quotation identiﬁed? e.g., participant number? | Page 8-12 |
| 30. Data and ﬁndings consistent | Was there consistency between the data presented and the ﬁndings? | Page 8 (Line 248-250)  **Yes** |
| 31. Clarity of major themes | Were major themes clearly presented in the ﬁndings? | Page 8 Table 2  **Yes** |
| 32. Clarity of minor themes | Is there a description of diverse cases or discussion of minor themes? | Page 8-12  **Yes** |
